# Supplementary material for: The potential health impact and healthcare cost savings of different sodium reduction strategies in Canada
Source: BMC Public Health. 2025 Jul 3;25:2379. doi: 10.1186/s12889-025-22941-8 (PMC12224586; doi:10.1186/s12889-025-22941-8)
Supplement: Supplementary file 1 — Supplementary Material 1. [file 12889_2025_22941_MOESM1_ESM.docx]

**Supplementary Material**

**The potential health impact and healthcare cost savings of different sodium reduction strategies in Canada**

Nadia Flexner^1,2^, Amanda C. Jones^3^, Ben Amies-Cull^4^, Linda Cobiac^5^, Eduardo Nilson^6,7^, Mary R. L’Abbe^1*^

^1^Department of Nutritional Sciences, Temerty Faculty of Medicine, University of Toronto, Toronto, Canada.

^2^Global Health Advocacy Incubator, Washington D.C., USA.

^3^Department of Public Health, University of Otago, Wellington, New Zealand.

^4^Nuffield Department of Primary Care Healthcare Sciences, University of Oxford, Oxford, United Kingdom.

^5^Griffith University, Queensland, Australia.

^6^Center for Epidemiological Research in Nutrition and Public Health, University of São Paulo, São Paulo, Brazil.

^7^Oswaldo Cruz Foundation (Fiocruz/Brasilia), Brasilia, Brazil.

^*^Corresponding authors: [mary.labbe@utoronto.ca](mailto:mary.labbe@utoronto.ca)

**Contents**

[**Table S1.** Structure of the PRIMEtime model 2](#_Toc191816715)

[**Table S2.** Disease data sources and processing description for the PRIMEtime Salt model 3](#_Toc191816716)

[**Table S3.** Extrapolation of disease data for older age groups 4](#_Toc191816717)

[**Table S4.** Intervention effects by DRI age/sex group, Canadian adults (≥19 y) 5](#_Toc191816718)

[**Table S5.** Direct healthcare costs: data sources and processing of health care costs inputs for the Canadian PRIMEtime Salt model^*^ 6](#_Toc191816719)

[**Table S6.** Direct healthcare costs (CAD 2019) inputs for the PRIMEtime Salt model^4^ 7](#_Toc191816720)

[**Table S7.** Relative risks of diseases associated with high sodium intake 7](#_Toc191816721)

[**Table S8.** Sodium-SBP parameters considered for the PRIMEtime Salt model 8](#_Toc191816722)

[**Table S9.** Disease-specific utility weights^8^ 8](#_Toc191816723)

[**Table S10.** Parameters for calculating background utility weights^8^ 8](#_Toc191816724)

[**Table S11.** Estimated health gains and healthcare cost savings over the lifetime of the cohort using 1.5%, 0% and 3% discount rate 9](#_Toc191816725)

[**Table S12.** Estimated health gains and healthcare cost savings over the lifetime of the cohort and at 10- and 50-year time horizons 10](#_Toc191816726)

# **Table S1.** Structure of the PRIMEtime model


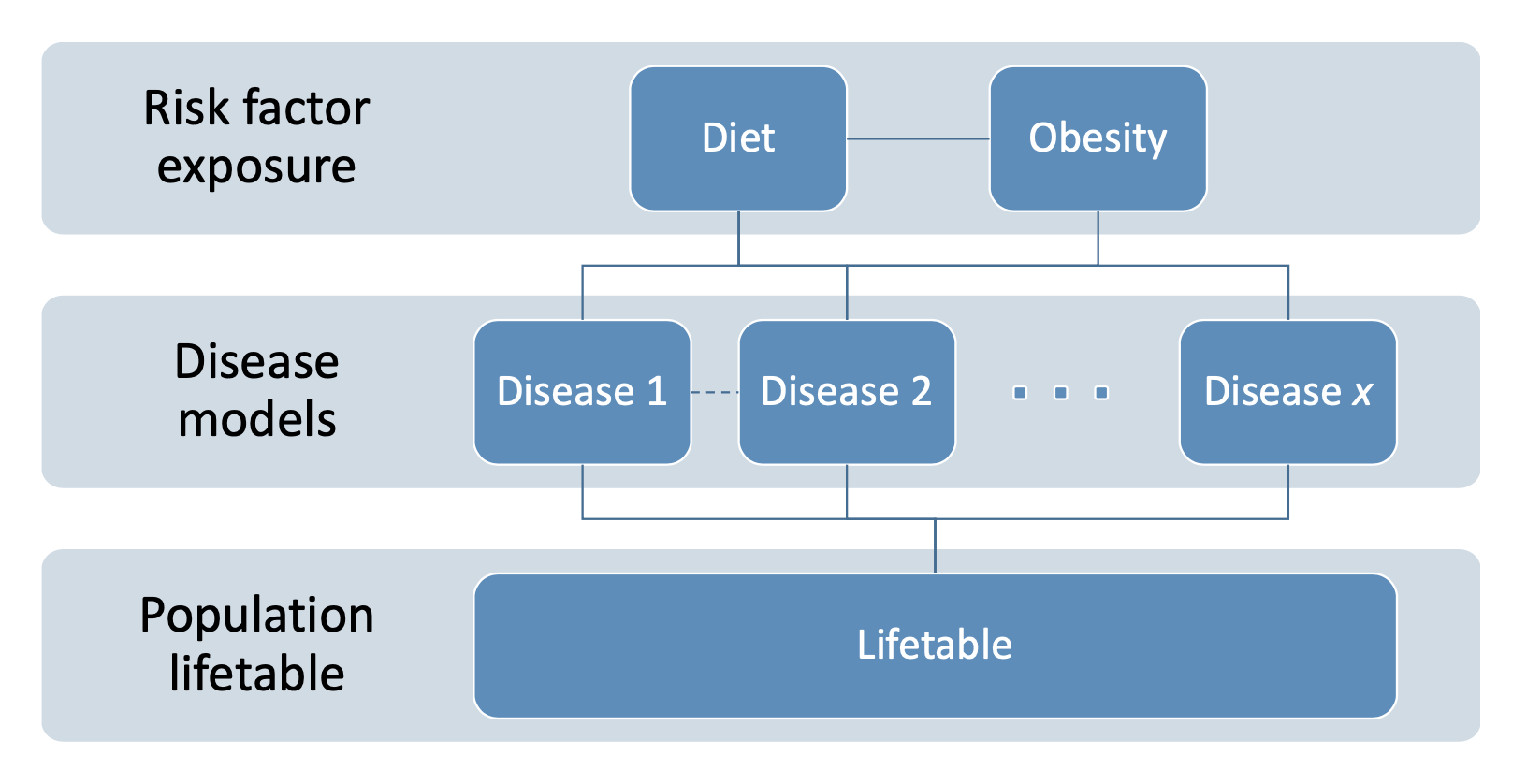


Figure from, Cobiac LJ, Law C, Scarborough P. PRIMEtime: an epidemiological model for informing diet and obesity policy. medRxiv. 2022:2022.2005. 2018.22275284^1^.

# **Table S2.** Disease data sources and processing description for the PRIMEtime Salt model

| **Diseases**  **(ICD-10 code)** | **Data sources** | **Pre-disbayes processing** | **disbayes estimations** |
| --- | --- | --- | --- |
| Ischemic heart disease (I20-I25) | Incidence cases: CCDSS (2019)  Disease-specific deaths: CANSIM Table 13-10-0147-01 (2019)  Prevalent cases: CCDSS (2019)  Remission: Assumed as 0 | Incidence, mortality, and prevalence rates were calculated using 2019 population estimates. Data was available in five-year age/sex groups up to age 90+ for all parameters. Rates were extrapolated to age 100+ using a polynomial trend line. Health data estimates were interpolated to one-year groups using a temporal disaggregation method to obtain smooth disaggregated counts, while maintaining the aggregated total. | The disbayes optimization method was used to estimate case fatality rates. It was assumed that case fatality was constant for all ages below 35. |
| Stroke  (I60-I69) | Incidence cases: CCDSS (2019)  Disease-specific deaths: CANSIM Table 13-10-0147-01 (2019)  Prevalent cases: CCDSS (2019)  Remission: Assumed as 0 | Incidence, mortality, and prevalence rates were calculated using 2019 population estimates. Data was available in five-year age groups up to age 90+ for all parameters. Rates were extrapolated to age 100+ using a polynomial trend line. Health data estimates were interpolated to one-year groups using a temporal disaggregation method to obtain smooth disaggregated counts, while maintaining the aggregated total. | The disbayes optimization method was used to estimate case fatality rates. It was assumed that case fatality was constant for all ages below 35. |

Abbreviations: CANSIM = Canadian Socio-economic Information Management System; CCDSS = Canadian Chronic Disease Surveillance System; GBD = Global Burden of Disease.

# **Table S3.** Extrapolation of disease data for older age groups

| **Disease**  **(ICD-10 code)** | **Measure** | **Polynomial order** |
| --- | --- | --- |
| Ischemic heart disease  (I20-I25) | Incidence-Female | Extrapolated using a **2nd order** polynomial trend line |
|  | Prevalence-Female | Extrapolated using a **2nd order** polynomial trend line |
|  | Deaths-Female | Extrapolated using a **4th order** polynomial trend line |
|  | Incidence-Male | Extrapolated using a **5th order** polynomial trend line |
|  | Prevalence-Male | Extrapolated using a **2nd order** polynomial trend line |
|  | Deaths-Male | Extrapolated using a **5th order** polynomial trend line |
| Stroke  (I60-I69) | Incidence-Female | Extrapolated using a **2nd order** polynomial trend line |
|  | Prevalence-Female | Extrapolated using a **3rd order** polynomial trend line |
|  | Deaths-Female | Extrapolated using a **4th order** polynomial trend line |
|  | Incidence-Male | Extrapolated using a **2nd order** polynomial trend line |
|  | Prevalence-Male | Extrapolated using a **3rd order** polynomial trend line |
|  | Deaths-Male | Extrapolated using a **3rd order** polynomial trend line |

# **Table S4.** Intervention effects by DRI age/sex group, Canadian adults (≥19 y)

| ***Salt (g/d)*** | | | | | | | | | | |
| --- | --- | --- | --- | --- | --- | --- | --- | --- | --- | --- |
| *Interventions* | *Meeting sodium intake recommendations* | | | *FOPL – food and beverage purchases* | | | *FOPL – food substitution* | | | |
| *Scenarios* | *S1 ∆* | *S2 ∆* | *S3 ∆* | *S4 ∆* | *S5 ∆* | *S6 ∆* | *S7 ∆* | *S8 ∆* | *S9 ∆* | *S10 ∆* |
| ***Total 19+*** | **-3.14** | **-1.89** | **-1.15** | **-0.32** | **-0.44** | **-0.53** | **-0.18** | **-0.33** | **-0.45** | **-0.65** |
| ***Males*** | | | | | | | | | | |
| 19-30 y | -3.40 | -2.05 | -1.25 | -0.40 | -0.55 | -0.67 | -0.20 | -0.38 | -0.54 | -0.73 |
| 31-50 y | -3.46 | -2.09 | -1.27 | -0.38 | -0.52 | -0.63 | -0.21 | -0.38 | -0.53 | -0.74 |
| 51-70 y | -3.54 | -2.13 | -1.30 | -0.35 | -0.47 | -0.58 | -0.21 | -0.38 | -0.52 | -0.75 |
| > 70 y | -3.64 | -2.20 | -1.33 | -0.32 | -0.44 | -0.54 | -0.22 | -0.38 | -0.52 | -0.76 |
| ***Females*** | | | | | | | | | | |
| 19-30 y | -2.55 | -1.54 | -0.92 | -0.29 | -0.39 | -0.48 | -0.14 | -0.27 | -0.37 | -0.52 |
| 31-50 y | -2.66 | -1.60 | -0.96 | -0.28 | -0.38 | -0.46 | -0.15 | -0.27 | -0.38 | -0.55 |
| 51-70 y | -2.81 | -1.69 | -1.02 | -0.27 | -0.36 | -0.44 | -0.16 | -0.28 | -0.38 | -0.57 |
| > 70 y | -2.88 | -1.73 | -1.04 | -0.25 | -0.34 | -0.41 | -0.16 | -0.28 | -0.38 | -0.58 |

Baseline and counterfactual scenarios were estimated using *CCHS-Nutrition 2015* ^2,3^*.* Baseline and counterfactual scenarios are described in methods. Usual intakes were estimated using the National Cancer Institute (NCI) method, and analyses were adjusted for age, sex, dietary misreporting status, weekend/weekday, and sequence of dietary recall. d, day; g, grams; FOPL, front-of-pack labeling; S1, scenario 1; S2, scenario 2; S3, scenario 3; S4, scenario 4; S5, scenario 5; S6, scenario 6; S7, scenario 7; S8, scenario 8; S9, scenario 9; S10, scenario 10.

# **Table S5.** Direct healthcare costs: data sources and processing of health care costs inputs for the Canadian PRIMEtime Salt model^*^

| **Diseases**  **(ICD-10 code)** | **Data sources** | **Methods** | **Direct costs^*^ (CAD 2019)** | **Disease**  **cases** |
| --- | --- | --- | --- | --- |
| Ischemic heart disease  (I20-I25) | Costs: Economic Burden of Illness in Canada 2010  Prevalent cases: CCDSS (2010)  Inflation factor (2010 to 2019): Consumer Price Index | The most suitable EBIC category for IHD were angina pectoris (903), acute myocardial infarction (904), and other ischemic heart diseases (905), defined by ICD codes I20, I21-I22, and I23-I25. To calculate the cost per case for IHD, the number of prevalent cases was obtained from the CCDSS 2010 database, corresponding to ICD codes I20, I21, I22, I23, I24, and I25 (data available for ≥ 20 years). Very close alignment between EBIC and cases data. | $3,955,629,248 | 2,275,810 |
| Stroke  (I60-I69) | Costs: Economic Burden of Illness in Canada 2010  Prevalent cases: CCDSS (2010)  Inflation factor (2010 to 2019): Consumer Price Index | The most suitable EBIC category for stroke were cerebral infarction (909), subarachnoid haemorrhage (910), intracerebral haemorrhage (911), acute but ill-defined stroke (912), and other ccerebrovascular diseases (913), defined by ICD codes I60, I61, I62, I63, I64, and I65-I69. To calculate the cost per case for stroke, the number of prevalent cases was obtained from the CCDSS 2010 database, corresponding to ICD codes G08, G45.x (exclude G45.4), H34.0, H34.1, I60.x, I61.x, I62.9, I63.x, I64, and I67.6. (Data available for ≥ 20 years). Close alignment between EBIC and cases data. | $1,536,400,337 | 715,980 |

^*^Direct costs estimations included attributable (drug, hospital care - day surgery, hospital care – inpatient, hospital care - other ambulatory care, hospital care - outpatient – clinic, hospital care - outpatient - emergency, and physician care) and unattributable direct cost (other institutions, other professionals, capital, public health, administration, and other health spending). Health care costs were inflated to 2019 CAD using the ‘health care’ sub-index from the Statistics Canada Consumer Price Index.

Abbreviations: CAD = Canadian Dollars; CANSIM = Canadian Socio-economic Information Management System; CCDSS = Canadian Chronic Disease Surveillance System.

# **Table S6.** Direct healthcare costs (CAD 2019) inputs for the PRIMEtime Salt model^4^

| ***Sex*** | ***Age*** | ***IHD*** | ***Stroke*** |
| --- | --- | --- | --- |
|  |  | $/prevalence case | $/prevalence case |
| Males | <55 | 3,768 | 3,867 |
|  | 55–64 | 2,726 | 2,678 |
|  | 65–74 | 2,183 | 2,336 |
|  | 75+ | 1,627 | 2,206 |
| Females | <55 | 1,829 | 3,085 |
|  | 55–64 | 1,516 | 2,064 |
|  | 65–74 | 1,384 | 2,128 |
|  | 75+ | 1,049 | 2,022 |

CAD, Canadian Dollars; IHD, ischemic heart disease.

# **Table S7.** Relative risks of diseases associated with high sodium intake

| ***Disease*** | ***Subgroup*** | ***Unit of change*** | ***Distribution*** | ***Relative risk (95% CI)*** |
| --- | --- | --- | --- | --- |
| IHD**^5^** | <49 | Per 20 mmHg of SBP | Lognormal | 0.49 (0.45–0.53) |
|  | 50–59 |  |  | 0.50 (0.49–0.52) |
|  | 60–69 |  |  | 0.54 (0.53–0.55) |
|  | 70-79 |  |  | 0.60 (0.58–0.61) |
|  | 79+ |  |  | 0.67 (0.64–0.70) |
| Stroke**^5^** | <49 | Per 20 mmHg of SBP | Lognormal | 0.36 (0.32–0.40) |
|  | 50–59 |  |  | 0.38 (0.35–0.40) |
|  | 60–69 |  |  | 0.43 (0.41–0.45) |
|  | 70–79 |  |  | 0.50 (0.48–0.52) |
|  | 79+ |  |  | 0.67 (0.63–0.71) |

IHD, ischemic heart disease; SBP, systolic blood pressure.

# **Table S8.** Sodium-SBP parameters considered for the PRIMEtime Salt model

| ***Parameter*** | ***Unit of change*** | ***Distribution*** | ***Mean (95% CI)*** |
| --- | --- | --- | --- |
| Theoretical minimum risk exposure level^6^ |  | Normal | 115 (mmHg) |
| Sodium-SBP dose response^7^ | Per 100 mmol/24 h urinary sodium | Normal | 5.80 (2.50–9.20) |

IHD, ischemic heart disease; SBP, systolic blood pressure.

# **Table S9.** Disease-specific utility weights^8^

| ***Disease*** | ***Mean utility (SD)*** |
| --- | --- |
| IHD incidence | -0.0625727 (0.0131711) |
| IHD prevalence | -0.0367975 (0.0257359) |
| Stroke incidence | -0.1170501 (0.0121435) |
| Stroke prevalence | -0.0731964 (0.0243687) |

IHD, ischemic heart disease; SD, standard deviation.

# **Table S10.** Parameters for calculating background utility weights^8^

| ***Parameter*** | ***Mean utility (SD)*** |
| --- | --- |
| Male | 0.0010046 (0.0006241) |
| Age 10-19 | 0.913 (0.0045) |
| Age 20-29 | 0.905 (0.0021) |
| Age 30-39 | 0.879 (0.0021) |
| Age 40-49 | 0.837 (0.0028) |
| Age 50-59 | 0.798 (0.0035) |
| Age 60-69 | 0.774 (0.0039) |
| Age 70-79 | 0.723 (0.0049) |
| Age 80-89 | 0.657 (0.0075) |

SD, standard deviation.

# **Table S11.** Estimated health gains and healthcare cost savings over the lifetime of the cohort using 1.5%, 0% and 3% discount rate

| **Counterfactual scenarios** | | Discount rate | **Males** | | | | **Females** | | | | **Totals** | | | |
| --- | --- | --- | --- | --- | --- | --- | --- | --- | --- | --- | --- | --- | --- | --- |
|  |  |  | Healthcare cost savings | ∆% | QALYs | ∆% | Healthcare cost savings | ∆% | QALYs | ∆% | Healthcare cost savings | ∆% | QALYs | ∆% |
| Meeting sodium intake recommendations | S1: Meeting AI sodium intake recommendations | **1.5%** | **(7,295,278,735)** |  | **425,297** |  | **(3,329,017,861)** |  | **276,295** |  | **(10,624,296,596)** |  | **701,592** |  |
|  |  | 0.0% | (12,641,343,057) | +73% | 814,916 | +92% | (5,998,122,784) | +80% | 539,317 | +95% | (18,639,465,841) | +75% | 1,354,233 | +93% |
|  |  | 3.0% | (4,517,328,555) | -38% | 240,235 | -44% | (1,992,136,932) | -40% | 154,319 | -44% | (6,509,465,487) | -39% | 394,554 | -44% |
|  | S2: Meeting WHO sodium intake recommendations | **1.5%** | **(4,625,515,221)** |  | **269,816** |  | **(2,066,782,422)** |  | **171,659** |  | **(6,692,297,643)** |  | **441,475** |  |
|  |  | 0.0% | (8,230,734,835) | +78% | 530,847 | +97% | (3,820,273,871) | +85% | 343,600 | +100% | (12,051,008,706) | +80% | 874,447 | +98% |
|  |  | 3.0% | (2,859,010,106) | -38% | 151,642 | -44% | (1,237,036,244) | -40% | 95,560 | -44% | (4,096,046,351) | -39% | 247,202 | -44% |
|  | S3: Meeting sodium reduction targets for packaged foods | **1.5%** | **(2,925,954,126)** |  | **169,889** |  | **(1,285,696,866)** |  | **106,296** |  | **(4,211,650,992)** |  | **276,185** |  |
|  |  | 0.0% | (5,096,725,339) | +74% | 327,012 | +92% | (2,319,253,143) | +80% | 207,594 | +95% | (7,415,978,482) | +76% | 534,607 | +94% |
|  |  | 3.0% | (1,783,063,843) | -39% | 94,674 | -44% | (756,641,270) | -41% | 58,544 | -45% | (2,539,705,113) | -40% | 153,218 | -45% |
| FOPL – food and beverage purchases | S4: Changes in food & beverage purchases (Chilean experience) | **1.5%** | **(813,641,779)** |  | **46,889** |  | **(335,637,085)** |  | **27,609** |  | **(1,149,278,864)** |  | **74,499** |  |
|  |  | 0.0% | (1,417,651,539) | +74% | 90,760 | +94% | (612,879,946) | +83% | 54,744 | +98% | (2,030,531,485) | +77% | 145,504 | +95% |
|  |  | 3.0% | (501,562,631) | -38% | 26,259 | -44% | (201,315,675) | -40% | 15,391 | -44% | (702,878,307) | -39% | 41,651 | -44% |
|  | S5: Based on WHO criteria to estimate cost-effectiveness of FOPL policies | **1.5%** | **(1,085,186,464)** |  | **62,782** |  | **(449,443,260)** |  | **37,084** |  | **(1,534,629,724)** |  | **99,867** |  |
|  |  | 0.0% | (1,898,498,196) | +75% | 121,255 | +93% | (824,236,776) | +83% | 73,476 | +98% | (2,722,734,972) | +77% | 194,731 | +95% |
|  |  | 3.0% | (671,324,288) | -38% | 35,178 | -44% | (267,611,069) | -40% | 20,513 | -45% | (938,935,357) | -39% | 55,691 | -44% |
|  | S6: Based on a FOPL systematic review and network meta-analysis | **1.5%** | **(1,328,579,013)** |  | **76,734** |  | **(547,635,403)** |  | **45,083** |  | **(1,876,214,416)** |  | **121,817** |  |
|  |  | 0.0% | (2,346,824,800) | +77% | 149,911 | +95% | (1,002,733,018) | +83% | 89,428 | +98% | (3,349,557,818) | +79% | 239,339 | +96% |
|  |  | 3.0% | (834,131,665) | -37% | 43,896 | -43% | (330,988,479) | -40% | 25,431 | -44% | (1,165,120,144) | -38% | 69,326 | -43% |
| FOPL – food substitution | S7: Based on food substitution for 30% of CCHS-Nutrition 2015 adult participants | **1.5%** | **(491,547,370)** |  | **28,620** |  | **(203,615,573)** |  | **16,872** |  | **(695,162,943)** |  | **45,492** |  |
|  |  | 0.0% | (858,895,440) | +75% | 55,335 | +93% | (366,705,990) | +80% | 32,916 | +95% | (1,225,601,430) | +76% | 88,251 | +94% |
|  |  | 3.0% | (301,245,041) | -39% | 16,019 | -44% | (122,321,118) | -40% | 9,456 | -44% | (423,566,159) | -39% | 25,475 | -44% |
|  | S8: Based on food substitution for 50% of CCHS-Nutrition 2015 adult participants | **1.5%** | **(874,780,208)** |  | **50,867** |  | **(355,922,614)** |  | **29,454** |  | **(1,230,702,822)** |  | **80,321** |  |
|  |  | 0.0% | (1,543,759,064) | +76% | 99,489 | +96% | (649,892,577) | +83% | 58,427 | +98% | (2,193,651,641) | +78% | 157,916 | +97% |
|  |  | 3.0% | (541,817,368) | -38% | 28,621 | -44% | (212,557,680) | -40% | 16,373 | -44% | (754,375,048) | -39% | 44,995 | -44% |
|  | S9: Based on food substitution for 70% of CCHS-Nutrition 2015 adult participants | **1.5%** | **(1,202,230,947)** |  | **69,860** |  | **(485,893,213)** |  | **40,203** |  | **(1,688,124,160)** |  | **110,063** |  |
|  |  | 0.0% | (2,096,511,836) | +74% | 134,818 | +93% | (873,921,045) | +80% | 78,352 | +95% | (2,970,432,881) | +76% | 213,170 | +94% |
|  |  | 3.0% | (738,225,183) | -39% | 39,011 | -44% | (288,227,575) | -41% | 22,216 | -45% | (1,026,452,759) | -39% | 61,227 | -44% |
|  | S10: Based on food substitution for all CCHS-Nutrition 2015 adult participants | **1.5%** | **(1,696,208,370)** |  | **98,309** |  | **(718,589,131)** |  | **59,318** |  | **(2,414,797,501)** |  | **157,628** |  |
|  |  | 0.0% | (3,025,892,309) | +78% | 194,991 | +98% | (1,331,225,632) | +85% | 119,714 | +102% | (4,357,117,941) | +80% | 314,705 | +100% |
|  |  | 3.0% | (1,073,018,334) | -37% | 56,854 | -42% | (438,533,336) | -39% | 33,856 | -43% | (1,511,551,669) | -37% | 90,710 | -42% |

# **Table S12.** Estimated health gains and healthcare cost savings over the lifetime of the cohort and at 10- and 50-year time horizons

| **Counterfactual scenarios** | | Time horizon | **Males** | | | | **Females** | | | | **Totals** | | | |
| --- | --- | --- | --- | --- | --- | --- | --- | --- | --- | --- | --- | --- | --- | --- |
|  |  |  | Healthcare cost savings | % | QALYs | % | Healthcare cost savings | % | QALYs | % | Healthcare cost savings | % | QALYs | % |
| Meeting sodium intake recommendations | S1: Meeting AI sodium intake recommendations | **Lifetime** | **(7,295,278,735)** | **100%** | **425,297** | **100%** | **(3,329,017,861)** | **100%** | **276,295** | **100%** | **(10,624,296,596)** | **100%** | **701,592** | **100%** |
|  |  | 10y | (527,901,503) | 7% | 16,569 | 4% | (221,058,642) | 7% | 10,453 | 4% | (748,960,145) | 7% | 27,022 | 4% |
|  |  | 50y | (5,613,154,471) | 77% | 288,889 | 68% | (2,469,649,992) | 74% | 182,860 | 66% | (8,082,804,463) | 76% | 471,748 | 67% |
|  | S2: Meeting WHO sodium intake recommendations | **Lifetime** | **(4,625,515,221)** | **100%** | **269,816** | **100%** | **(2,066,782,422)** | **100%** | **171,659** | **100%** | **(6,692,297,643)** | **100%** | **441,475** | **100%** |
|  |  | 10y | (328,425,488) | 7% | 10,274 | 4% | (135,370,573) | 7% | 6,381 | 4% | (463,796,061) | 7% | 16,656 | 4% |
|  |  | 50y | (3,543,093,273) | 77% | 183,234 | 68% | (1,534,950,993) | 74% | 114,145 | 66% | (5,078,044,266) | 76% | 297,379 | 67% |
|  | S3: Meeting sodium reduction targets for packaged foods | **Lifetime** | **(2,925,954,126)** | **100%** | **169,889** | **100%** | **(1,285,696,866)** | **100%** | **106,296** | **100%** | **(4,211,650,992)** | **100%** | **276,185** | **100%** |
|  |  | 10y | (208,060,087) | 7% | 6,503 | 4% | (83,860,708) | 7% | 3,951 | 4% | (291,920,795) | 7% | 10,454 | 4% |
|  |  | 50y | (2,239,733,134) | 77% | 114,974 | 68% | (949,039,210) | 74% | 70,048 | 66% | (3,188,772,343) | 76% | 185,022 | 67% |
| FOPL – food and beverage purchases | S4: Changes in food & beverage purchases (Chilean experience) | **Lifetime** | **(813,641,779)** | **100%** | **46,889** | **100%** | **(335,637,085)** | **100%** | **27,609** | **100%** | **(1,149,278,864)** | **100%** | **74,499** | **100%** |
|  |  | 10y | (57,322,630) | 7% | 1,752 | 4% | (21,946,766) | 7% | 1,018 | 4% | (79,269,395) | 7% | 2,770 | 4% |
|  |  | 50y | (622,755,414) | 77% | 31,722 | 68% | (250,834,251) | 75% | 18,437 | 67% | (873,589,665) | 76% | 50,160 | 67% |
|  | S5: Based on WHO criteria to estimate cost-effectiveness of FOPL policies | **Lifetime** | **(1,085,186,464)** | **100%** | **62,782** | **100%** | **(449,443,260)** | **100%** | **37,084** | **100%** | **(1,534,629,724)** | **100%** | **99,867** | **100%** |
|  |  | 10y | (78,345,247) | 7% | 2,395 | 4% | (29,794,435) | 7% | 1,385 | 4% | (108,139,681) | 7% | 3,779 | 4% |
|  |  | 50y | (839,388,154) | 77% | 42,648 | 68% | (336,565,830) | 75% | 24,674 | 67% | (1,175,953,984) | 77% | 67,322 | 67% |
|  | S6: Based on a FOPL systematic review and network meta-analysis | **Lifetime** | **(1,328,579,013)** | **100%** | **76,734** | **100%** | **(547,635,403)** | **100%** | **45,083** | **100%** | **(1,876,214,416)** | **100%** | **121,817** | **100%** |
|  |  | 10y | (95,785,422) | 7% | 2,931 | 4% | (36,245,245) | 7% | 1,683 | 4% | (132,030,667) | 7% | 4,614 | 4% |
|  |  | 50y | (1,035,607,041) | 78% | 52,682 | 69% | (411,620,130) | 75% | 30,163 | 67% | (1,447,227,171) | 77% | 82,845 | 68% |
| FOPL – food substitution | S7: Based on food substitution for 30% of CCHS-Nutrition 2015 adult participants | **Lifetime** | **(491,547,370)** | **100%** | **28,620** | **100%** | **(203,615,573)** | **100%** | **16,872** | **100%** | **(695,162,943)** | **100%** | **45,492** | **100%** |
|  |  | 10y | (35,668,871) | 7% | 1,115 | 4% | (13,347,584) | 7% | 628 | 4% | (49,016,455) | 7% | 1,743 | 4% |
|  |  | 50y | (378,362,463) | 77% | 19,391 | 68% | (151,335,914) | 74% | 11,175 | 66% | (529,698,378) | 76% | 30,566 | 67% |
|  | S8: Based on food substitution for 50% of CCHS-Nutrition 2015 adult participants | **Lifetime** | **(874,780,208)** | **100%** | **50,867** | **100%** | **(355,922,614)** | **100%** | **29,454** | **100%** | **(1,230,702,822)** | **100%** | **80,321** | **100%** |
|  |  | 10y | (62,468,463) | 7% | 1,941 | 4% | (23,437,511) | 7% | 1,100 | 4% | (85,905,974) | 7% | 3,041 | 4% |
|  |  | 50y | (682,324,948) | 78% | 34,913 | 69% | (266,640,396) | 75% | 19,652 | 67% | (948,965,344) | 77% | 54,566 | 68% |
|  | S9: Based on food substitution for 70% of CCHS-Nutrition 2015 adult participants | **Lifetime** | **(1,202,230,947)** | **100%** | **69,860** | **100%** | **(485,893,213)** | **100%** | **40,203** | **100%** | **(1,688,124,160)** | **100%** | **110,063** | **100%** |
|  |  | 10y | (86,343,814) | 7% | 2,681 | 4% | (32,037,421) | 7% | 1,502 | 4% | (118,381,235) | 7% | 4,183 | 4% |
|  |  | 50y | (920,944,627) | 77% | 47,337 | 68% | (360,614,380) | 74% | 26,662 | 66% | (1,281,559,007) | 76% | 73,999 | 67% |
|  | S10: Based on food substitution for all CCHS-Nutrition 2015 adult participants | **Lifetime** | **(1,696,208,370)** | **100%** | **98,309** | **100%** | **(718,589,131)** | **100%** | **59,318** | **100%** | **(2,414,797,501)** | **100%** | **157,628** | **100%** |
|  |  | 10y | (122,570,274) | 7% | 3,823 | 4% | (47,732,138) | 7% | 2,245 | 4% | (170,302,412) | 7% | 6,069 | 4% |
|  |  | 50y | (1,318,282,218) | 78% | 67,710 | 69% | (538,887,184) | 75% | 39,829 | 67% | (1,857,169,402) | 77% | 107,539 | 68% |

**References**

1. Cobiac LJ, Law C, Scarborough P. PRIMEtime: an epidemiological model for informing diet and obesity policy. *medRxiv*. 2022:2022.05. 18.22275284.

2. Statistics Canada. Canadian Community Health Survey – Nutrition: Public Use Microdata File. Accessed October 12, 2022. <https://www150.statcan.gc.ca/n1/en/catalogue/82M0024X>

3. Health Canada. 2015 Canadian Community Health Survey - Nutrition. Reference Guide to Understanding and Using the Data. Accessed October 12, 2022. <https://www.canada.ca/en/health-canada/services/food-nutrition/food-nutrition-surveillance/health-nutrition-surveys/canadian-community-health-survey-cchs/reference-guide-understanding-using-data-2015.html>

4. Public Health Agency of Canada. Economic Burden of Illness in Canada, 2010. Accessed March 15, 2023. <https://www.canada.ca/en/public-health/services/publications/science-research-data/economic-burden-illness-canada-2010.html>

5. Lewington S, Clarke R, Qizilbash N, Peto R, Collins R. Age-specific relevance of usual blood pressure to vascular mortality: a meta-analysis of individual data for one million adults in 61 prospective studies. *The Lancet*. 2002;360(9349):1903-1913.

6. Danaei G, Ding EL, Mozaffarian D, et al. The Preventable Causes of Death in the United States: Comparative Risk Assessment of Dietary, Lifestyle, and Metabolic Risk Factors. *PLOS Medicine*. 2009;6(4):e1000058. doi:10.1371/journal.pmed.1000058

7. He FJ, Li J, MacGregor GA. Effect of longer term modest salt reduction on blood pressure: Cochrane systematic review and meta-analysis of randomised trials. *Bmj*. 2013;346

8. Sullivan PW, Slejko JF, Sculpher MJ, Ghushchyan V. Catalogue of EQ-5D scores for the United Kingdom. *Medical Decision Making*. 2011;31(6):800-804.
